# Enrichment of human osteosarcoma stem cells based on hTERT transcriptional activity

**DOI:** 10.18632/oncotarget.1554

**Published:** 2013-11-05

**Authors:** Ling Yu, Shiqing Liu, Chun Zhang, Bo Zhang, Bruno M. Simões, Rachel Eyre, Yi Liang, Huichao Yan, Zheng Wu, Weichun Guo, Robert B. Clarke

**Affiliations:** ^1^ Department of Orthopedics, Renmin Hospital of Wuhan University, Jiefang Road, Wuhan, Hubei, P.R. China; ^2^ Institute of Cancer Sciences, University of Manchester, Wilmslow Road, Manchester, UK; ^3^ State Key Laboratory of Oncology in Southern China and the Department of Experimental Research, Sun Yat-Sen University Cancer Center, Dongfeng Road, Guangzhou, Guangdong, P.R. China; ^4^ Opening Laboratory for Oversea Scientists, Wuhan University School of Basic Medical Science, Donghu Road, Wuhan, Hubei, P.R. China; ^5^ Department of Radiation Oncology, Tumor Hospital Xiangya School of Medicine of Central South University, Tongzipo Road, Changsha, Hunan, P.R.China

**Keywords:** Osteosarcoma, Heterogeneity, Cancer stem cells, Telomerase, Metastasis, Drug resistance

## Abstract

Telomerase is crucial for the maintenance of stem/progenitor cells in adult tissues and is detected in most malignant cancers, including osteosarcoma. However, the relationship between telomerase expression and cancer stem cells remains unknown. We observed that sphere-derived osteosarcoma cells had higher telomerase activity, indicating that telomerase activity might be enriched in osteosarcoma stem cells. We sorted subpopulations with high or low telomerase activity (TEL) using hTERT transcriptional promoter-induced green fluorescent protein (GFP). The TEL^pos^ cells showed an increased sphere and tumor propagating capacity compared to TEL^neg^ cells, and enhanced stem cell-like properties such as invasiveness, metastatic activity and resistance to chemotherapeutic agents both *in vitro* and *in vivo*. Furthermore, the telomerase inhibitor MST312 prevented tumorigenic potential both *in vitro* and *in vivo*, preferentially targeting the TEL^pos^ cells. These data support telomerase inhibition as a potential targeted therapy for osteosarcoma stem-like cells.

## INTRODUCTION

Osteosarcoma is an extremely aggressive form of bone cancer which has a peak incidence in the second decade of life [1, 2]. Prior to systemic chemotherapy amputation was the only choice of treatment, and the high rate of pulmonary micrometastasis makes 5 year survival very low [3]. For the last 4 decades, neoadjuvant chemotherapy and limb salvage surgery have greatly improved both the survival and life quality of the patient [4]. However, despite intensive chemotherapy, the survival rate for high-grade osteosarcoma has improved little and almost 40% of osteosarcoma patients die of their disease [5].

Osteosarcoma cells within tumor tissue demonstrate significant heterogeneity with respect to tumorigenic and metastatic potential [6, 7]. The biological basis underlying this cellular heterogeneity has potential therapeutic implications. In contrast to the stochastic model, the cancer stem cell model predicts that heterogeneity within a tumor is derived from a hierarchal organization [8-11]. Cancer stem cells (CSCs) are thus thought to share important properties with normal tissue stem cells such as self-renewal and differentiation. In addition, cancer stem cells have been proposed to be responsible for metastasis and resistance to anticancer drugs [12, 13]. Therefore, the inadequacy of current treatments may result from the inability to effectively target the cancer stem cells within osteosarcoma.

Properties of normal stem cells have been widely used to identify cancer stem cells. There are several methods used to isolate the osteosarcoma stem cells, including cell surface markers [14, 15], Hoechst effluxing (SP) [16], label retention (PKH26) [17], the Aldefluor assay [18] and promoter reporter assays [19]. For example, the mesenchymal stem cell markers CD117 and Stro-1 have been successfully applied to isolate osteosarcoma initiating cells associated with metastasis and drug resistance [14]. However, these methods have not identified a way to target these tumorigenic cellular subpopulations.

Human telomerase is a reverse transcriptase enzyme composed of a catalytic component, telomerase reverse-transcriptase (TERT) and a telomerase RNA component (TERC) [20]. In most human normal somatic cells, telomerase activity is undetectable, however, stem/progenitor cells in self-renewing tissues express telomerase. In addition, telomerase activity is detected in most malignant cancers, including osteosarcoma [21]. Given the important role of telomeres and telomerase in stem cells and cancer [22-24], we predicted a high expression of telomerase in cancer stem cells, and aimed to investigate its role in the cancer stem cell phenotype.

To test this, we stably transduced osteosarcoma cell lines with a lentiviral vector in which the human TERT promoter drives expression of green fluorescent protein (GFP). Based on high activity of the fluorescent reporter, we isolated a distinct subpopulation of cells with tumor-initiating properties from the human osteosarcoma cell lines. These cells show high drug-resistant and metastatic properties, and furthermore we demonstrate that telomerase inhibition is effective for eradicating these osteosarcoma stem-like cells.

## RESULTS

### Osteosarcoma sphere cells are enriched for high telomerase activity

**Figure 1:**
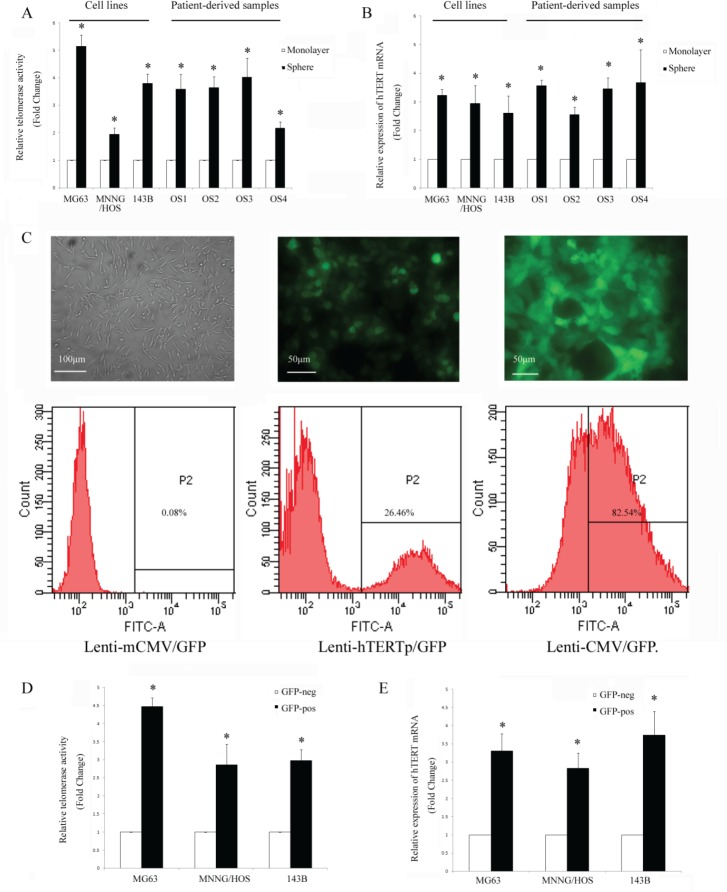
Osteosarcoma cells are heterogeneous according to their telomerase activity (A) Telomerase activity was detected by TERP-ELISA, and it was higher in sphere cultured cells. (B) hTERT mRNA level (determined by qPCR) was upregulated in sphere-cultured cells. *P<0.01 compared with the monolayer. (C) Representative images of fluorescence of MG63 cells transduced with different GFP lentiviruses (left: negative control, mCMV-GFP, 100×; middle: hTERT-GFP, 200× and right: positive control, CMV-GFP, all without puromycin selection, 200×). The proportion of GFP positive cells were analyzed using flow cytometry. (D) The osteosarcoma cells were sorted according to their GFP status. Telomerase activity was increased in GFP positive cells and (E) hTERT mRNA level was also higher in GFP positive cells. *P<0.01 compared with the GFP negative cells.

A proportion of osteosarcoma cells are capable of forming spheres when cultured in a serum free, anchorage-independent environment [25]. Spheres show high levels of staining for the osteosarcoma stem cell markers Stro-1 and CD117, and sphere-forming cells have greater capacity to initiate tumors, which supports their increased stem cell content [14]. To test whether stem cells have higher telomerase activity, we used the TRAP-ELISA assay and analysed hTERT mRNA expression level in the cells in different culture conditions. We found telomerase activity to be much higher in sphere cultured cells than monolayer cultured cells both in osteosarcoma cell lines and primary osteosarcoma cells, with an average fold change of 3.15±0.34 (*P*<0.01) (Fig. 1A). In accordance with the telomerase activity, the expression level of hTERT mRNA was also upregulated under sphere culture condition, with an average fold change of 3.47±1.10 (*P*<0.01) (Fig. 1B).

### A subset of osteosarcoma cells can activate an exogenous hTERT Promoter and GFP expression

In order to perform live tracing of the telomerase activity, we constructed a lentiviral hTERTpromoter-GFP reporter vector with a puromycin selection cassette, and established osteosarcoma cell lines stably expressing the hTERT promoter reporter. To assess the validity of the lentivirus, we used both a transcriptionally inactive negative control virus and a transcriptionally active positive control virus (Fig. 1C). The negative control group had no GFP positive cells, which demonstrate the specificity of the cloned hTERT promoter reporter. The positive control vector, without puromycin selection, demonstrated high numbers of GFP positive cells implying that the osteosarcoma cell lines are easily infected by this lentiviral vector. We found that only a subset of osteosarcoma cells can activate the exogenous hTERT promoter and express GFP (Fig. 1C). The GFP positive portion of MG63, MNNG/HOS and 143B were 23.24±3.88, 15.51±2.79 and 22.44±2.74, respectively. We then asked whether hTERT transcription activity correlates with hTERT mRNA and telomerase activity in human osteosarcoma cell lines. We divided the osteosarcoma cells into GFP-pos and GFP-neg groups according to their GFP expression, and found that both the telomerase activity and hTERT mRNA levels were higher in the GFP-pos cells, with the average fold increase of 3.42±0.89 (*P*<0.01) and 3.29±0.45 (*P*<0.01), respectively (Fig. 1D-E).

### Osteosarcoma cells with high telomerase promoter activity express cancer stem cell markers and related genes

**Figure 2:**
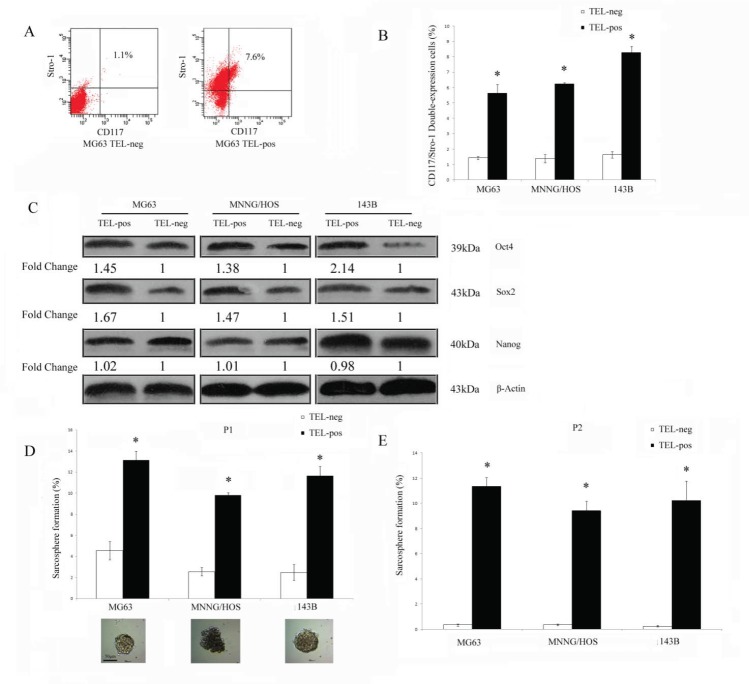
TEL^pos^ osteosarcoma cells show increased stem cell-like properties *in vitro* (A) A representative FACS analysis of CD117/Stro-1 double positive cells in TEL^pos^ and TEL^neg^ MG63 cells. (B) TEL^pos^ cells had an increased CD117 and Stro-1 double positive cell population. *P<0.01 compared with the TEL^neg^ cells. (C) TEL^pos^ cells had upregulated expression of Sox2 and Oct4, but not Nanog. Fold changes were calculated from 3 independent experiments normalised using β-actin expression (see also Supplementary Table 2). (D) Sphere culture of osteosarcoma cells. The ability to form sarcospheres was higher in TEL^pos^ cells at the first passage (P1). *P<0.01 compared with the TEL^neg^ cells. Representative images of sarcosphere are shown, scale bar was 50 μm. (E) The ability of TEL^neg^ cells cells to form sarcospheres was almost depleted at the secondary passage (P2). *P<0.01 compared with the TEL^neg^ cells.

Next, we separated three osteosarcoma cell lines into TEL^pos^ and TEL^neg^ subpopulations according to their GFP expression status in order to compare their stem cell-like properties. CD117 and Stro-1 are linked to cancer stem cells with metastatic and drug resistant properties in osteosarcoma [14], therefore we investigated if TEL^pos^ cells were enriched with cells expressing these markers. The CD117 and Stro-1 double positive rate for MG63, MNNG/HOS and 143B TEL^pos^ cells were 5.62±0.75, 6.24±0.09 and 8.27±0.41, respectively. Across the three cell lines tested, we observed an average of 4.51±0.55 fold higher of double positive cells in TEL^pos^ cells compared with TEL^neg^ cells (*P*<0.01) (Fig. 2A-B). As a control, the Stro-1 and CD117 status of the non-transduced parental MG63 cells was also assessed (Supplementary Fig.1A). CSCs are reported to possess a transcriptional profile similar to that of embryonic stem cells (ES cells) [19], therefore we investigated if there is differential expression of these genes between TEL^pos^ and TEL^neg^ cells. We found that Sox2 and Oct4 were greatly upregulated in GFP-positive cells, whereas Nanog was expressed in both GFP-positive and negative cells without significant difference (Fig. 2C) (Detailed densitometry data is shown in Supplementary Table 2).

### Osteosarcoma cells possessing high telomerase activity have increased tumorigenic potential both *in vitro* and *in vivo*

Sarcosphere culture is an *in vitro* system used to evaluate the ability of osteosarcoma stem-like cells to form sphere colonies in suspension and whether they self-renew in secondary culture. We compared the ability of TEL^pos^ and TEL^neg^ cells to form primary and secondary sarcospheres. TEL^pos^ cells formed more sarcospheres than TEL^neg^ cells, with an average fold increase of 3.8±0.9 (Fig. 2D). Significantly, when dissociated sphere cells were plated for a second generation of sphere culture, self-renewal from TEL^neg^ spheres was almost depleted, whereas cells from spheres grown from TEL^pos^ cells underwent self-renewal very efficiently (Fig.2E).

**Figure 3:**
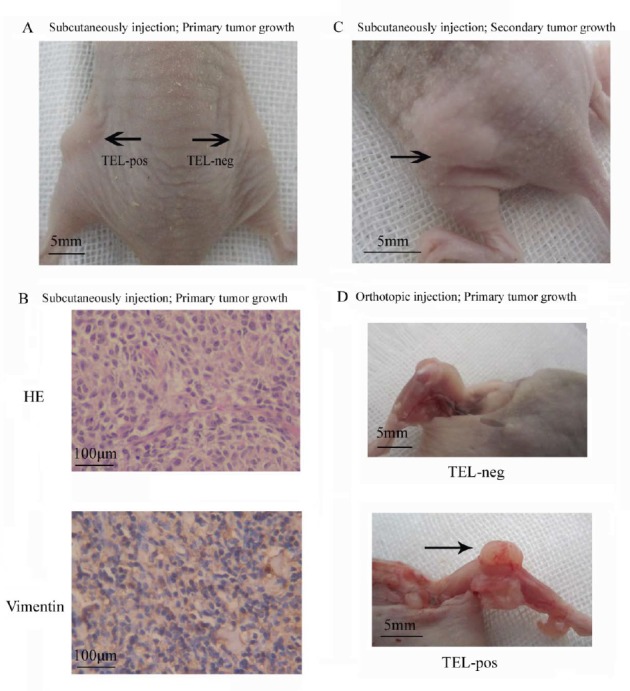
TEL^pos^ osteosarcoma cells show increased stem cell-like properties *in vivo* (A) MG63 TEL^pos^ cells show an increased capacity to form tumors *in vivo* by subcutaneous injection. The image represents the relative tumorigenic potential of 5×10^3^ TEL^pos^ compared with 5×10^3^ TEL^neg^ cells. (B) Representative H and E and vimentin staining of MG63 TEL^pos^ cells derived tumor (100×). (C) MG63 TEL^pos^ cells derived from xenografts form tumor after serial transplantation. (D) MNNG/HOS TEL^pos^ cells show an increased capacity to form tumors *in vivo* by orthotopic injection. The pictures shown the relative tumorigenic potential of 5×10^3^ TEL^neg^ compared with 5×10^3^ TEL^pos^ cells.

The most stringent test of CSC activity is their ability to initiate tumors. We therefore subcutaneously injected serial dilutions of TEL^pos^ and TEL^neg^ MG63 cells into immunocompromised mice and examined the rate of tumor formation over a period of 6 months. As shown in Table 1, the majority of mice (7/8) injected with 5,000 TEL^pos^ cells formed tumors, whereas only one in 8 mice injected with 5×10^4^ TEL^neg^ cells showed tumor formation. The extreme limiting dilution assay (ELDA) calculation estimated a 374-fold increase in cancer stem cell frequency in TEL^pos^ compared to TEL^neg^ cells (Fig. 3A; Table 1). Tumors were further analysed by histological examination, and expression of vimentin indicated their mesenchymal origin (Fig. 3B). Furthermore, we isolated TEL^pos^ cells from two different MG63 derived tumors and serially transplanted these into further mice. Tumor formation was observed in 83.3% (5/6) of mice (n = 6) injected with 5,000 cells (Fig. 3C). Serial transplantability of TEL^pos^ cells confirmed their *in vivo* self-renewal activity. We next tested the ability of TEL^pos^ cells to initiate osteosarcomas in the bone niche using MNNG/HOS cells. Mice were injected orthotopically into the tibia with TEL^pos^ or TEL^neg^ cells. 6 out of 8 mice injected with 5,000 TEL^pos^ cells formed tumors, whereas no tumours were formed in mice injected with TEL^neg^ cells, even when 5×10^4^ cells were injected. ELDA analysis indicated a 232-fold increase in tumour-initiating cell frequencies in TEL^pos^ compared to TEL^neg^ cells (Fig. 3D; Table 1).

**Table 1:** Tumor forming ability following subcutaneous and orthotopic injections

Cell line	MG63 (s.c.)		*P*-value	MNNG/HOS (orthotopic)		*P*-value
Cell number	TEL^neg^	TEL^pos^		TEL^neg^	TEL^pos^
5,000	0/8	7/8	0.001	0/8	6/8	0.003
50,000	1/8	8/8	0.001	0/8	8/8	0.000
500,000	3/8	8/8	0.013	4/8	8/8	0.038
ELDA (95% CI)^a^	1/898899 (1/2455639- 1/329046)	1/2404 (1/5807- 1/996)	0.000	1/835203 (1/2222499- 1/313865)	1/3607 (1/8572- 1/1518)	0.000

a ELDA: Extreme limiting dilution analysis; CI: confidence interval.

### Osteosarcoma cells with high telomerase activity have multipotency

**Figure 4:**
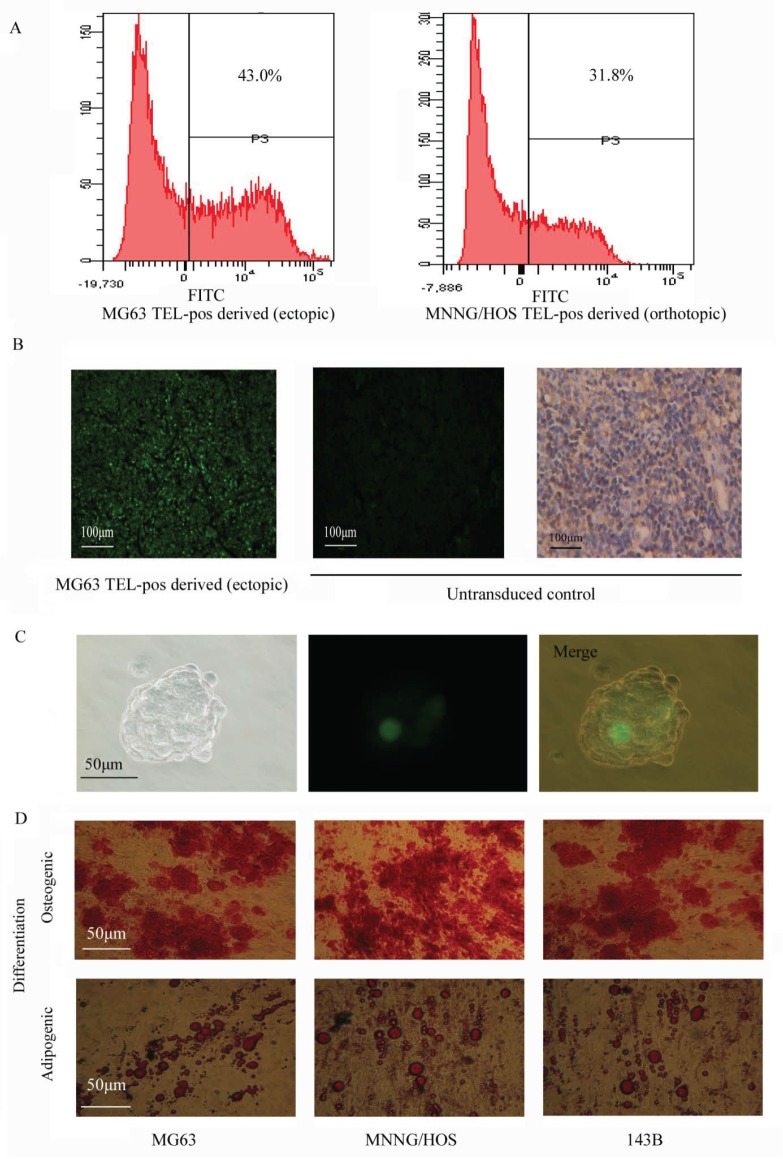
Multipotency of the TEL^pos^ cells (A) Tumor tissues derived from TEL^pos^ cells were dissociated into single cells to analyze the GFP expression, which showed the production of TEL^neg^ cells by TEL^pos^ cells. (B) Left: A representative fluorescence image of MG63 TEL^pos^-derived tumor section was shown (100×); middle: non-transduced cells was set as negative control (100×); right: non-transduced cells stained with anti-human MHC Class I antibody (100×). (C) *In vitro* differentiation of TEL^pos^ cells into TEL^neg^, a representative clonally derived sphere of MG63 is shown (400×). (D) *In vitro* differentiation of TEL^pos^ cells, a representative image of TEL^pos^ cell differentiation from three osteosarcoma cell lines (200×).

Many cancer stem cell types possess the capability of multipotent differentiation [14, 26]. We demonstrated that cells recovered from TEL^pos^ xenograft tumors could be re-sorted into GFP-enriched and non-GFP subpopulations (Fig. 4A). This implies that TEL^pos^ cells can differentiate into TEL^neg^ cells *in vivo*, and was confirmed in a TEL^pos^-derived tumor section, where both GFP-positive and GFP-negative cells were observed. To exclude the possibility of contamination from mouse derived-tissue, xenograft tumors were stained with anti-human MHC class I Antibody (Santa Cruz sc-32235), which does not cross-react with mouse tissue. It was found that almost 100% of the cells stained positive, indicating that they were human-derived (Fig. 4B). We also observed that a single TEL^pos^ cell could form a sarcosphere colony consisting of TEL^pos^ and TEL^neg^ cells, suggesting *in vitro* differentiation of TEL^pos^ cells into TEL^neg^ cells (Fig.4C).

It is not common to see the differentiation of regular osteosarcoma cells along osteogenic or adipogenic lineage, and therefore this method can be used to test the multipotency of osteosarcoma stem cells. We observed that TEL^pos^ cells were able to undergo osteogenic and adipogenic differentiation *in vitro*, whilst TELneg cells could not be induced along these lineages. These data indicate the multipotency of TEL^pos^ cells (Fig. 4D, Supplementary Fig.1B).

### Osteosarcoma cells with high telomerase activity show increased invasion *in vitro*, metastasis *in vivo* and drug resistance

**Figure 5:**
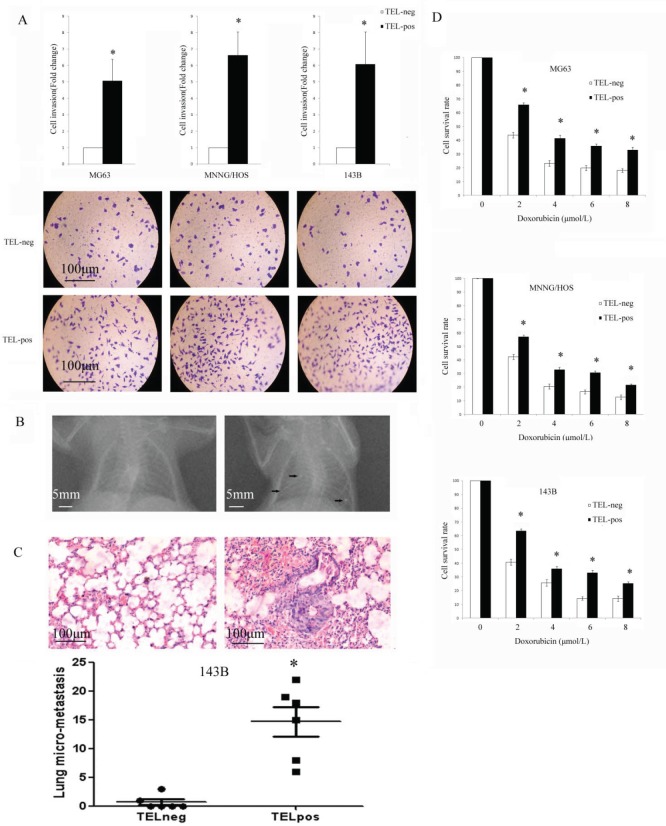
TEL^pos^ cells show higher invasion, metastasis potential and drug resistance (A) The invasive potential of TEL^pos^ cells was higher. *P<0.01 compared with the TEL^neg^ cells. Representative images of invasion of each cell line were shown (100×). (B) TEL^pos^ 143B cells injected *in vivo* produce obvious detected pulmonary nodules by X-ray examination. (C) The histology examination of 143B cell lung micrometastases. TEL^pos^ 143B cells produce a higher number of pulmonary micrometastatic lesions. *P<0.01compared with the TEL^neg^ cells. (D) Cell sub-populations were treated with increasing concentrations of doxorubicin for 24 h. TEL^pos^ cells showed an increased viability in all three cell lines. *P<0.01compared with the TEL^neg^ cells.

We performed a Matrigel Transwell invasion assay to evaluate the invasive properties of different cells *in vitro*. TEL^pos^ cells showed a nearly 3-fold higher invasive potential compared with that of TEL^neg^ cells (Fig. 5A). To investigate the metastatic potential of TEL^pos^ and TEL^neg^ cells, we used the 143B cell line due to its relatively high tendency to metastasise *in vivo*. We orthotopically injected 1x10^5^ 143B cells and the development of pulmonary metastasis was monitored by X-ray detection. Overall, the mice showed a 75% (6/8) appearance rate of lung nodules following TEL^pos^ cells injection, whereas none of the TEL^neg^ injected mice showed obvious lung nodules (Fig. 5B). We also quantified micrometastatic lesions by serial lung section (Fig.5C), which further confirmed the higher metastatic potential of TEL^pos^ cells than TEL^neg^ cells.

We next examined whether telomerase active cells could survive current treatments, such as doxorubicin, which is one of the most widely used reagents for osteosarcoma chemotherapy. We found that in all three cell lines TEL^pos^ cells showed a higher resistance to doxorubicin than TEL^neg^ cells (Fig. 5D).

### Treatment with Telomerase inhibitor MST312 preferentially targets GFP positive CSCs

**Figure 6:**
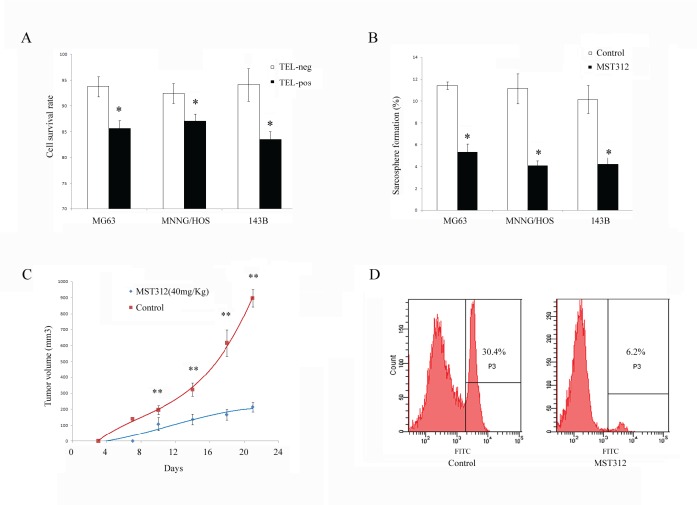
MST312 targets TEL^pos^ cells (A) Cell viability following MST312 treatment of MG63, MNNG/HOS, and 143B cells. Both TEL^pos^ and TEL^neg^ cells showed a decrease in cell viability but TEL^pos^ cells respond significantly more to the treatment (*P*<0.01). (B) *In vitro* sphere formation of TEL^pos^ cell was inhibited after exposure to MST312 (*P*<0.01). (C) MST312 inhibited *in vivo* tumor forming ability of TEL^pos^ MG63 cells (*P*<0.01). (D) TEL^pos^ cell population of xenografted tumors were decreased after MST312 treatment.

Next, we tested whether the telomerase inhibitor MST312 targets the osteosarcoma cells *in vitro*. We divided the cells into TEL^pos^ and TEL^neg^ subgroups and treated with MST312 for 96h. We found a decrease in the cell viability of both subgroups, but a significantly greater inhibitory effect was observed in TEL^pos^ cells (Fig. 6A). Furthermore, we found that MST312 inhibited *in vitro* sphere formation of TEL^pos^ cells, with an average inhibition rate of 58.3±5.1% (Fig. 6B). TEL^pos^ MG63 cells were then subcutaneously injected into nude mice, and the mice were treated with MST312. After 3 weeks the tumors in control mice were ~ 1cm^3^, while tumours in the MST312 treated mice were 5-fold smaller (Fig. 6C). We then analysed MG63-TEL^pos^ derived tumors treated with MST312 for the GFP positive cell population, and found it to be decreased from 27.3±3.0 to 7.9±2.2 (Fig. 6D).

## DISCUSSION

CSCs share many properties with normal stem cells, which are known to express telomerase. Telomerase is also found in 90% of human malignancies and has been reported to predict the outcome of osteosarcoma [27]. We thus proposed to use telomerase activity to isolate osteosarcoma stem cells.

First, we asked whether there was differential activity of telomerase between osteosarcoma stem cells and non-stem cells. Using anchorage-independent, serum-starved culture conditions, a subpopulation of cells capable of self-renewal can be enriched as spherical clones, termed sarcospheres [25]. We found that telomerase activity was markedly enhanced in sarcospheres compared to monolayer cells. This also applied to primary cells from telomerase-expressing osteosarcoma. These data support the theory that telomerase activity can be used to isolate osteosarcoma stem cells. We therefore used a hTERT promoter-GFP lentiviral reporter and established the stable reporter cell lines to assess the cellular telomerase activity. We confirmed that in the three cell lines used the cells can be divided into two subpopulations according to the hTERT promoter activity, which strongly correlated with the expression of the hTERT mRNA and telomerase activity. This enrichment was also observed in four primary telomerase-positive osteosarcomas.

We then tested whether hTERT promoter active cells possess CSC activities such as self-renewal and *in vivo* transplantation, and if our putative CSCs overlapped with other identified CSC populations in osteosarcoma. The TEL^pos^ cells possessed a much stronger capacity to form spheroids than TEL^neg^ cells. This ability was not compromised after serial passage, which implies the property of self-renewal. Cell surface markers such as CD117 and Stro-1 have previously been successfully used in isolating osteosarcoma stem cells, and we assessed expression of these in TEL^pos^ cells. Phenotypic characterization of TEL^pos^ cells showed that they selectively expressed CD117 and Stro-1. Previous studies have reported that ES cell genes play a crucial role in CSCs [29, 30] and TEL^pos^ cells were found to overexpress both embryonic stem cell (ES cell)-specific transcription factors Oct4 and Sox2, although Nanog was expressed in both TEL^pos^ and TEL^neg^ cells without significant difference.

Using a limited dilution xenograft assay, we confirmed that the sub-population capable of activating the hTERTpromoter/GFP reporter exhibited significantly enhanced tumorigenic activity *in vivo*. Following either orthotopic or ectopic transplantation, these TEL^pos^ cells consistently formed tumors at a lower number of injected cells, and these tumors were able to initiate new tumors after serial transplantation. A further key property of cancer stem cells is the ability to give rise to heterogeneous tumors comprised of tumorigenic and non-tumorigenic cell populations. TEL^pos^ cells differentiated toward several mesenchymal phenotypes *in vitro*, including osteogenic and adipogenic differentiation. Furthermore, TEL^pos^cells could produce GFP negative cells both *in vitro* and *in vivo*, providing evidence that these cells can give rise to phenotypically diverse progeny.

CSCs have been shown to be more capable of metastasis [31, 32]. Previous research has demonstrated CD117^+^Stro-1^+^ osteosarcoma cells to be highly metastatic and enriched for a metastasis-associated marker CXCR4 [14]. In addition, it has been shown that osteosarcoma cells capable of activating a transgenic Oct-4 promoter were more likely to form metastases in the lung [19]. Consistent with these studies, our study established that TEL^pos^ cells had higher invasive and metastatic properties compared to TEL^neg^ cells, *in vitro* and *in vivo*, respectively. TERT has been reported to promote epithelial-mesenchymal transition in gastric cancer cells [33]. It is thus feasible that TEL^pos^ osteosarcoma could utilise EMT-related genes to promote metastasis.

Many factors contribute to the drug resistance of CSCs [34, 35]. We found that TEL^pos^ cells exhibited a higher resistance to doxorubicin treatment, demonstrating their drug-resistant capability. Doxorubicin's mechanism of action is via intercalating DNA, causing DDR (DNA damage response) and activating apoptosis [36, 37]. There is increasing evidence that telomerase also participates in DNA repair, and this effect is independent of telomere elongation [38-40]. We speculate that high hTERT expression confers cells with enhanced DNA repair capacity, which results in resistance to doxorubicin.

Different strategies targeting telomerase are currently being tested in several cancers, including osteosarcoma [41, 42]. However, few studies have analyzed whether these therapies are preferentially effective in CSCs. MST312 is a small molecule that has emerged as a promising candidate for anti-telomeric therapy, and it has been reported to be effective in ALDH+ lung cancer stem cells [43, 44]. In our study, we have shown that MST312 has a stronger inhibitory effect on TEL^pos^ than TEL^neg^ cells. It can inhibit sphere formation *in vitro* and hinder the *in vivo* tumor formation of TEL^pos^ cells, and it decreases the percentage of TEL^pos^ cells in xenografted tumors. These findings provide evidence that a telomerase inhibitor can be preferentially effective in CSCs. It is reported that TERT promotes epithelial proliferation through Myc- and Wnt-related pathways [45], and the Wnt pathway has also been shown to be involved in osteosarcoma pathogenesis [46, 47]. Due to the facts that (i): The MST312 dose used in this study was nontoxic [44]; and (ii): the short duration of treatment, we believe that the effects observed here are mostly due to inhibition of proliferation and self-renewal, and independent of telomere-maintenance.

In contrast to methods using cell surface markers, we found that TEL^pos^ cells appear to constitute a relatively large proportion in the tumor mass. This is in accordance with previous research in which osteosarcoma stem cells were isolated using an Oct4/GFP reporter [19]. These data suggest that osteosarcoma contain a large percentage of tumor-initiating cells. Osteosarcoma presents as pleomorphic, malignant, spindle cells that secrete osteoid and express surface antigens commonly associated with mesenchymal stem cells (MSCs), such as CD29, CD44, CD56, CD90 and CD105 [48]. Given these observations, it is likely that osteosarcoma is derived from mesenchymal stem cells. However, it is reported that mesenchymal stem cell have no or very low levels of telomerase activity [50]. In addition, osteosarcoma cell lines such as U2OS and Saos2 [51] and 2/3 of clinical osteosarcoma are telomerase negative [27, 52]. It is thus reasonable that our strategy might not be suitable for those patients where telomerase activity and hTERT expression are undetectable.

Our study is the first to show that osteosarcomas contain a subpopulation of cells identified by high telomerase activity, which possess stem cell-like properties including self-renewal, multi-potency, pulmonary metastatic potential and drug resistance. Further, this study identifies telomerase inhibition as a potential candidate for osteosarcoma stem cell-targeted therapy, which could help to solve the most lethal characteristics of osteosarcoma—recurrence, metastasis and chemotherapy resistance.

## METHODS

### Primary osteosarcoma cells and cell lines

Primary osteosarcoma samples were retrieved from four patients with conventional high-grade osteosarcoma, and were confirmed to be telomerase expressing. Primary osteosarcoma cell lines (OS1-4) were established from tumor lesions taken from surgical biopsies. All the samples were obtained with informed consent, and the study protocol was approved by the institutional review board of Renmin Hospital of Wuhan University. None of specimens had been exposed to chemotherapy or radiotherapy, the diagnosis was confirmed by the histological analysis, and the clinicopathological features are described in Supplementary Table 1. Tumor tissues were processed directly after biopsy. In brief, tumors were washed, cut into small pieces and digested at 37˚C with 0.25% trypsin and 0.1% type II collagenase for 30 min and 2 h, respectively. Cells isolated after digestion were plated out into subsequent culture. The telomerase expressing human osteosarcoma cell lines MG63and MNNG/HOS were purchased from the Shanghai Institute for Biological Sciences of the Chinese Academy of Sciences. 143B was obtained from China Center for Type Culture Collection (CCTCC). All the cells were cultured in RPMI-1640 containing 10% FBS and 1% penicillin/streptomycin. Cells were propagated in a humidified environment at 37˚C with 5% CO_2_ and 100% humidity. Cell viability was determined using trypan blue staining.

### Generation of hTERT-GFP reporter osteosarcoma cell line

The hTERT promoter region (1.5 kb) [53-55]was synthesized and cloned into the multiple cloning site of the lentiviral transcriptional reporter vector pGreenFire1-mCMV-EF1-Puro (TR010PA-P; System Biosciences, Mountain View, CA), with ClaI and EcoRI recognistion site on 5' and 3' end, respectively. The promoter region sequence was confirmed by Shanghai Sangon Biotech Co. Ltd. We transfected HEK293-T cells with the constructs and obtained the viral supernatants. To generate stably expressing reporter cell lines, we infected osteosarcoma cells with the viral supernatant and selected the cells with puromycin (5μg/ml) for one week. The transcriptionally inactive pGreenFire-mCMV (TR010PA-P) was used as a negative control and the transcriptionally active pGreenFire-CMV (TR011PA-1) was used as a positive control.

### FACScan analysis and sorting

The hTERT-GFP reporter osteosarcoma cells were harvested with fresh 0.25% trypsin solution (Sigma-Aldrich, Inc.) and resuspended in PBS. Cells were maintained on ice until analysis. The GFP expression was assessed and cells were sorted into TEL^pos^ and TEL^neg^ subpopulation according to their GFP expression status, using a Becton-Dickinson FACSort (San Jose, CA, USA). For analysis of cell surface markers, the cells were harvested and resuspended in PBS/0.5% normal rabbit serum (Sigma-Aldrich, Inc.), and blocked on ice for 15 min. Cells were subsequently labelled with Alexa Fluor® 647 anti-human Stro-1 antibody (BioLegend) and/or PE anti-human CD117 (c-kit) antibody (BioLegend) for 60 min and maintained on ice until analysis. The expression was assessed by flow cytometry and data were analyzed using WinMDI software (Scripps Research Institute, La Jolla, CA, USA).

### TRAP-ELISA assay

Telomerase activity was determined using the TeloTAGGG PCR ELISA^PLUS^ kit (Roche, Mannheim, Germany) according to the manufacturer's protocol. In brief, cells were cultured in monolayer or sphere condition for 14 days and then harvested. 2×10^5^ cells per sample were lysed in 200 μL lysis buffer, and 2 μL cell extract was used for the TRAP reaction. Telomeric repeats were added to biotinylated synthetic primers by telomerase, the elongation product was then amplified by PCR. PCR products were then immobilized to a streptavidin-coated microplate. The immobilized PCR products were detected by an anti-digoxigenin antibody, and then visualized by peroxidase. The absorbance of the samples was measured at 450 nm. Heat-treated cell extract was used as the negative control. A template DNA with the same sequence as a telomerase product with 8 telomeric repeats was used as a positive control. Relative telomerase activities (RTA) were calculated according to protocol.

### Drug sensitivity assessment

Osteosarcoma cells (TEL^pos^ and TEL^neg^) were cultured for 2 days in complete media to allow cells to recover from sorting stress. Cells (5,000 per well) were cultured in 96-well plates for 1 day and then treated with increasing concentrations of doxorubicin (0-10.0 μmol/L) for 24 hours. Cell viability was measured by CCK8 assay.

### Matrigel invasion assay

*In vitro* invasion assays were performed in transwell chambers (Costar, Cambridge, MA). The upper side of the porous polycarbonate membrane (8.0 μm pore size) was coated with 10 μg/cm^2^ reconstituted Matrigel basement membrane (Sigma). Cells (2×10^5^/well) were seeded on the upper side of the filter and incubated in RPMI-1640 medium with 0.5% FBS. The lower chamber was filled with complete culture medium containing 10% FBS. After 24h, cells migrated to the lower side were fixed with 4% paraformaldehyde and stained with 0.1% crystal violet. The filters were photographed and cells were counted.

### Tumor spheroid assay

The sphere formation assay followed procedures previously described [25]. In brief, cells were plated in six-well ultralow attachment plates (Corning Inc., Corning, NY) at a density of 5,000 cells/well in RPMI-1640 supplemented with B27 Supplement (Invitrogen), 10 ng/mL human EGF (Sigma-Aldrich), and 10 ng/mL human bFGF (Sigma-Aldrich). Cells were incubated at 37˚C in a humidified atmosphere of 95% air and 5% CO_2_. Fresh aliquots of EGF and bFGF were added every other day. After culture for 14 days, colonies larger than 50 μm in size were regarded as sarcospheres and quantitated by inverted phase contrast microscopy. The spheres were processed to form the next generation of spheres every 14 days.

### Animals and transplantation Assay

To determine the *in vivo* tumorigenicity, BALB/C nude mice about 6 weeks old were purchased from and maintained at the Wuhan University Center for Animal Experiment. The care and use of animals has been reviewed and approved by the Institutional Animal Care and Use Committee (IACUC) (approval number: 2011006). After sorting by FACS, live cells were counted by trypan blue staining, and suspended in 10μL of 50% Matrigel/PBS. The mice were randomly divided according to their injected cells and site. Tumors were grown by subcutaneous (MG63) or orthotopic (MNNG/HOS) inoculation with 5 × 10^3^ to 5 × 10^5^ cells. Tumor onset was set at 5 mm diameter, mice were monitored as long as 6 months.

To evaluate the potential to metastasise, we performed orthotopic injections of 143B cells. 1×10^5^ cells in 10μL of 50% Matrigel/PBS were injected into femoral bone marrow cavities of anesthetized BALB/C nude mice. Mice were monitored until 90 days after injection or until tumors reached 10 mm in diameter. At the end of observation point, the mice were anesthetized and X-ray images were taken to detect pulmonary metastases using the KODAK Digital Radiography System. The mice were then sacrificed. Tumor and lung samples were fixed in 4% paraformaldehyde and embedded in paraffin. Tissue sections were stained with haematoxylin and eosin. Data were accumulated from at least three independent experiments.

### Differentiation

Osteosarcoma cells were plated in 96-well plates. When cells reached confluency, they were cultured in osteogenic differentiation media for 3 weeks and then stained by alizarin red. For adipogenic differentiation, the cells were cultured in differentiation medium for 5 days. Cells were then fixed with 10% buffered formalin and stained with Oil Red O.

### Quantitative real-time PCR

Total RNA was isolated and reverse transcribed. Real-time PCR was then performed using an ABI 7900 System in the presence of SYBR- Green. The following gene-specific primers were used: hTERT (5' GGAGCAAGTTGCAAAGCATTG -3', 5'-TCCCACGACGTAGTCCATGTT-3') and GAPDH (5' AGAAGGCTGGGGCTCATTTG-3', 5'-AGGGGCCATCCACAGTCTTC-3'). Target sequences were amplified at 95˚C for 10 min, followed by 40 cycles of 95˚C for 15 s and 60˚C for 1 min. GAPDH was used as endogenous normalization control. All assays were performed in triplicate. The fold change in mRNAs expression was determined according to the method of 2^ΔΔCt^.

### Western Blot

Cell lysates were extracted using RIPA lysis buffer containing protease inhibitor cocktail. Protein concentrations were determined using BCA method. Cell lysates containing 50 μg of protein were loaded and separated on 10% SDS-PAGE gels and subsequently transferred to polyvinylidene difluoride membranes (PVDF). Membranes were blocked in 5% milk solution, incubated at 4˚C overnight with following primary antibodies: rabbit monoclonal Oct4 (Abcam; 1:1,000), rabbit polyclonal Sox2 (Abcam; 1: 1,000), rabbit polyclonal Nanog (Santa Cruz; 1: 2,000), mouse monoclonal β-actin (Santa Cruz; 1: 1,000). They were then washed, and incubated with horseradish peroxidase conjugated secondary antibody at dilution 1:5,000 for 1h at room temperature. Membranes were then washed and developed using ECL Substrate. Densitometry was performed using Image J software.

### Testing the efficiency of telomerase inhibitor MST312

Osteosarcoma cell lines were sorted and cultured as described above. The monolayer cultured cells were treated with MST312 for 72 h, with a final concentration of 1μM MST312. For sphere culture, fresh medium with MST312 was used every 3 days, and cultured for 14 days. Xenotransplatation assay was carried out as described above, and 5 × 10^6^ MG63 cells were injected subcutaneously into the flanks of the mice. A total of 8 mice were randomly divided into control (no treatment) and MST312-treated groups, with 4 in each. The MST312 was administered by intraperitoneal injection (40 mg/kg) every other day, starting after injection. Tumor volume was measured every 3-4 days according to the formula: V = length × (width)^2^/2. All mice were sacrificed 21 days after cell injection and tissues were kept for FACS analysis.

### Statistical analyses

Each experiment was performed independently at least three times. Fisher's Exact Test (one-sided) was used to determine the statistical significance of *in vivo* tumorigenesis. Otherwise, values were expressed as mean ± SD, and statistical significance (*P* < 0.05) was determined using Student's t test.

## Supplementary Figures and Tables




