# Supplementary material for: Enrichment of human osteosarcoma stem cells based on hTERT transcriptional activity
Source: Oncotarget. 2013 Nov 5;4(12):2326–38. doi: 10.18632/oncotarget.1554 (PMC3926830; doi:10.18632/oncotarget.1554)
Supplement: Supplementary file 1 [file oncotarget-04-2326-s001.pdf]

Enrichment of human osteosarcoma stem cells based on hTERT transcriptional activity - Yu et al

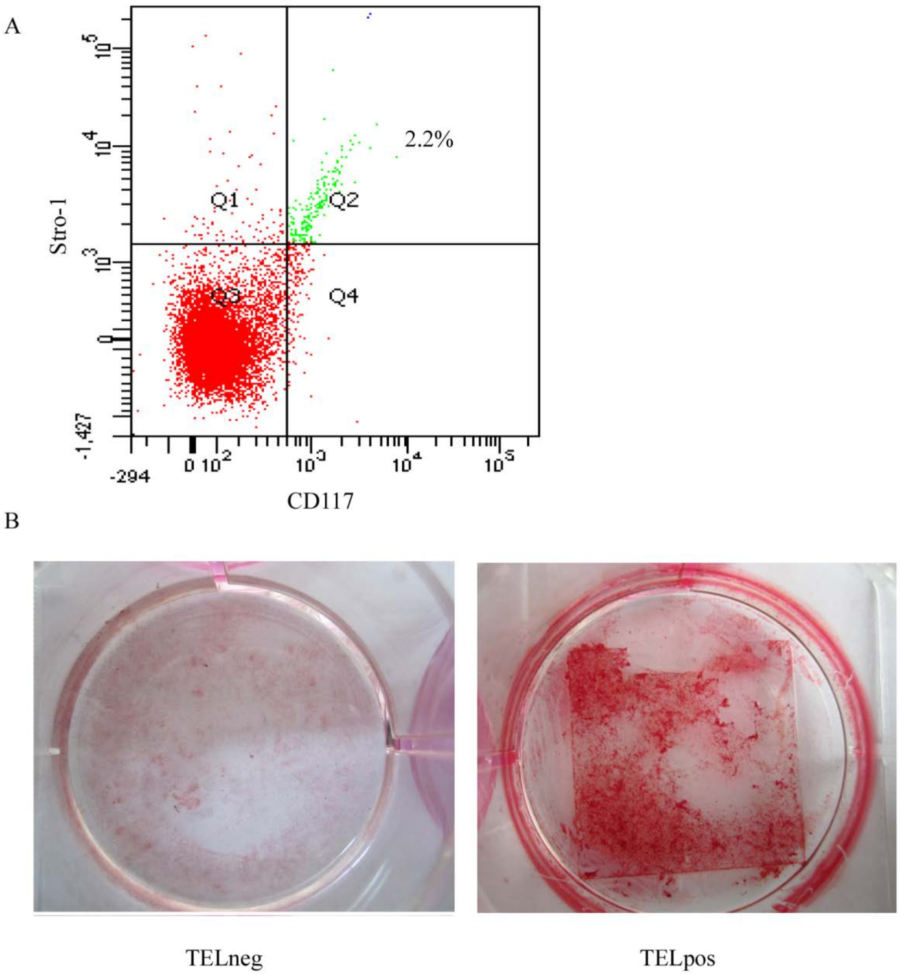

Supplementary Fig.1: (A) The Stro-1 and CD117 expression status of the non-transduced parental MG63 cells was shown as a control. (B) Gross observation of different MG63cells to undergo osteogenic differentiation in vitro. TELpos showed massive alizarin red stain, while we could not induce TELneg cells along osteogenic lineage.
