# Supplementary material for: Enrichment of human osteosarcoma stem cells based on hTERT transcriptional activity
Source: Oncotarget. 2013 Nov 5;4(12):2326–38. doi: 10.18632/oncotarget.1554 (PMC3926830; doi:10.18632/oncotarget.1554)
Supplement: Supplementary file 2 [file oncotarget-04-2326-s002.pdf]

Enrichment of human osteosarcoma stem cells based on hTERT transcriptional activity – Yu et al

Supplementary Table 1: Clinicopathologic features of patient derived samples.

	Gender	Age	Tumor Site	Histopathology	Histologic grade	Clinical stage	Treatment	Telomerase
OS1	Male	24	Femur	Osteoblastic	Poorly	IIB	No	+
OS2	Male	31	Humerus	Fibroblastic	Moderately	IB	No	+
OS3	Female	25	Femur	Osteoblastic	Poorly	IIA	No	+
OS4	Male	19	Tibia	Osteoblastic	Poorly	IIB	No	+

Supplementary Table 2: Densitometry data* of ES-related genes expression in three osteosarcoma cell lines

	MG63			MNNG/HOS			143B		
	TELpos	TELneg	P	TELpos	TELneg	P	TELpos	TELneg	P
Oct4	0.22± 0.03	0.15± 0.02	0.034	0.37± 0.04	0.26± 0.04	0.029	0.40± 0.04	0.18± 0.06	0.006
Sox2	0.27± 0.03	0.16± 0.06	0.038	0.38± 0.03	0.26± 0.04	0.016	0.30± 0.03	0.20± 0.02	0.007
Nanog	0.22± 0.03	0.22± 0.04	0.875	0.41± 0.03	0.40± 0.01	0.769	0.65± 0.08	0.66± 0.05	0.860

*The data was obtained from 3 independent experiments and normalised to β-actin expression.

Supplementary Data 1 Sequence of hTERT promoter region (1.5kb), with ClaI and EcoRI recognition site on 5’ and 3’ end, respectively

5’-ATCGATTAAAATTGTGTTTTCTATGTTGGCTTCTCTGCAGAGAACCAGTGTAAGCTACAACTTAACTTTTGTT
GGAACAAATTTTCCAAACCGCCCCTTTGCCCTAGTGGCAGAGACAATTCACAAACACAGCCCTTTAAAAAGGC
TTAGGGATCACTAAGGGGATTTCTAGAAGAGCGACCTGTAATCCTAAGTATTTACAAGACGAGGCTAACCTCC
AGCGAGCGTGACAGCCCAGGGAGGGTGCGAGGCCTGTTCAAATGCTAGCTCCATAAATAAAGCAATTTCCTC
CGGCAGTTTCTGAAAGTAGGAAAGGTTACATTTAAGGTTGCGTTTGTTAGCATTTCAGTGTTTGCCGACCTCA
GCTACAGCATCCCTGCAAGGCCTCGGGAGACCCAGAAGTTTCTCGCCCCTTAGATCCAAACTTGAGCAACCCG
GAGTCTGGATTCCTGGGAAGTCCTCAGCTGTCCTGCGGTTGTGCCGGGGCCCCAGGTCTGGAGGGGACCAGT
GGCCGTGTGGCTTCTACTGCTGGGCTGGAAGTCGGGCCTCCTAGCTCTGCAGTCCGAGGCTTGGAGCCAGGT
GCCTGGACCCCGAGGTTGCCCTCCACCCTGTGCGGGCGGGATGTGACCAGATGTTGGCCTCATCTGCCAGACA
GAGTGCCGGGGCCCAGGGTCAAGGCCGTTGTGGCTGGTGTGAGGCGCCCGGTGCGCGGCCAGCAGGAGCG
CCTGGCTCCATTTCCCACCCTTTCTCGACGGGACCGCCCCGGTGGGTGATTAACAGATTTGGGGTGGTTTGCTC
ATGGTGGGGACCCCTCGCCGCCTGAGAACCTGCAAAGAGAAATGACGGGCCTGTGTCAAGGAGCCCAAGTC
GCGGGGAAGTGTTGCAGGGAGGCACTCCGGGAGGTCCCGCGTGCCCGTCCAGGGAGCAATGCGTCCTCGGG
TTCGTCCCCAGCCGCGTCTACGCGCCTCCGTCCTCCCCTTCACGTCCGGCATTCGTGGTGCCCGGAGCCCGACG
CCCCGCGTCCGGACCTGGAGGCAGCCCTGGGTCTCCGGATCAGGCCAGCGGCCAAAGGGTCGCCGCACGCAC
CTGTTCCCAGGGCCTCCACATCATGGCCCCTCCCTCGGGTTACCCCACAGCCTAGGCCGATTCGACCTCTCTCC
GCTGGGGCCCTCGCTGGCGTCCCTGCACCCTGGGAGCGCGAGCGGCGCGCGGGCGGGGAAGCGCGGCCCA
GACCCCCGGGTCCGCCCGGAGCAGCTGCGCTGTCGGGGCCAGGCCGGGCTCCCAGTGGATTCGCGGGCACA
GACGCCCAGGACCGCGCTTCCCACGTGGCGGAGGGACTGGGGACCCGGGCACCCGTCCTGCCCCTTCACCTT
CCAGCTCCGCCTCCTCCGCGCGGACCCCGCCCCGTCCCGACCCCTCCCGGGTCCCCGGCCCAGCCCCCTCCGG
GCCCTCCCAGCCCCTCCCCTTCCTTTCCGCGGCCCCGCCCTCTCCTCGCGGCGCGAGTTGAATTC-3’
